# In Silico Identification of Peanut Peptides Suitable for Allergy Immunotherapy in HLA-DRB1*03:01-Restricted Patients

**DOI:** 10.3390/ph17081097

**Published:** 2024-08-21

**Authors:** Irini Doytchinova, Mariyana Atanasova, Stanislav Sotirov, Ivan Dimitrov

**Affiliations:** Drug Design and Bioinformatics Lab, Faculty of Pharmacy, Medical University of Sofia, 1000 Sofia, Bulgaria; matanasova@pharmfac.mu-sofia.bg (M.A.); 113660@students.mu-sofia.bg (S.S.); idimitrov@pharmfac.mu-sofia.bg (I.D.)

**Keywords:** logo method, HLA-DRB1*03:01, peptide binding prediction, peanut allergy, immunotherapy

## Abstract

Peanut allergy, a prevalent and potentially severe condition affecting millions worldwide, has been linked to specific human leukocyte antigens (HLAs), suggesting increased susceptibility. Employing an immunoinformatic strategy, we developed a “logo model” based on amino acid frequencies in the peptide binding core and used it to predict peptides originating from 28 known peanut allergens binding to HLA-DRB1*03:01, one of the susceptibility alleles. These peptides hold promise for immunotherapy in HLA-DRB1*03:01 carriers, offering reduced allergenicity compared to whole proteins. By targeting essential epitopes, immunotherapy can modulate immune responses with minimal risk of severe reactions. This precise approach could induce immune tolerance with fewer adverse effects, presenting a safer and more effective treatment for peanut allergy and other allergic conditions.

## 1. Introduction

A food allergy is a condition in which the immune system reacts abnormally to certain foods, triggering a range of mild symptoms like hives, itching, and swelling to more severe reactions like anaphylaxis, which can be life-threatening. Common food allergens originate from peanuts, tree nuts, shellfish, milk, eggs, wheat, and soy [1]. Diagnosis often involves skin prick tests or blood tests, and management typically requires the patient to strictly avoid the allergen and carry emergency medication like epinephrine [2]. 

Peanut allergy is one of the most common and potentially severe food allergies, affecting millions worldwide [3]. It occurs when the immune system mistakenly identifies the proteins in peanuts as harmful, triggering an allergic reaction. At present, 17 peanut allergens have been identified (http://www.allergen.org) [4]. Sensitization to Ara h 1, Ara h 2, and Ara h 3 has been detected in 57% to 90% of American peanut-allergic patients, in 37% to 74% of Swedish patients, and in 16% to 42% of Spanish patients [5]. Ara h 2 stands out as the predominant peanut allergen, with its specific IgE serving as the most effective serological marker for diagnosing peanut allergy [6]. On the other hand, Ara h 3 emerges as one of the principal allergenic proteins from peanuts, being recognized via serum IgE testing in approximately 45% of peanut-allergic patients [7]. Ara h 3 constitutes 19% of the total protein content in peanut extracts and is classified as a major peanut allergen. Despite ongoing research, there is currently no cure for peanut allergy, highlighting the importance of awareness, education, and preparedness in dealing with this condition.

The association between peanut allergy and the human leukocyte antigen (HLA) system, also known as human major histocompatibility complex (MHC) antigens, has been a subject of interest in allergy research [8,9,10,11,12,13,14]. HLAs bind to peptide fragments derived from pathogens or self-proteins and present them to T-cells for inspection. This process is pivotal for the activation of immune responses, including adaptive immunity. Variations in HLA genes influence individual susceptibility to diseases, transplantation compatibility, and vaccine efficacy. Studies have identified a potential link between peanut allergy and certain HLA alleles, such as HLA-DRB1*08 [8], DQB1*04 [8,13], DQB1*06:03 [13], and HLA-DRB1*03:01 [8,9,13]. Research suggests that individuals carrying these alleles may have an increased susceptibility to peanut allergy due to their immune response to specific peanut proteins. 

Allergy immunotherapy, also known as allergy shots or allergy desensitization, is a treatment aimed at reducing allergic reactions by gradually exposing the immune system to allergens. This therapy involves injecting increasing doses of allergens over time, allowing the body to build up a tolerance and reduce its allergic response. It is commonly used to treat allergies to pollen [15], dust mites [16], pet dander [17], and certain insect venoms [18]. Immunotherapy can alleviate symptoms and, in some cases, provide long-term relief. While it requires commitment and patience due to its gradual nature, allergy immunotherapy has been shown to be effective in reducing the severity of allergic reactions and improving the quality of life of many allergy sufferers.

Immunotherapy for peanut allergy aims to reduce allergic reactions upon accidental exposure to peanuts. Studies have shown promising results, with many participants experiencing an increase in their tolerance to peanuts [19,20,21,22,23,24,25,26]. However, immunotherapy carries risks, including potential allergic reactions during treatment. Its long-term efficacy and safety remain under investigation, but immunotherapy offers hope for individuals with peanut allergy, potentially providing a life-changing treatment option.

Here, we applied an immunoinformatic approach to derive a model for the prediction of peptides binding to HLA-DRB1*03:01, one of the HLA alleles susceptible to peanut allergy. As the model is based on amino acid frequencies in the peptide binding core, like the visual representation of amino acid sequence conservation in proteins (logo graphs) [27], we named it the “logo model” [28]. In the sequence logo, each position is depicted by a stack of letters, in which the size of the letters indicates their frequency within the sequences. Next, we used the derived logo model to identify the high-affinity binding peptides among 28 known peanut allergens [4]. We hypothesize that these high-affinity binders are suitable for the immunotherapy of patients carrying HLA-DRB1*03:01. Peptides used for immunotherapy offer a promising alternative to whole proteins due to their reduced allergenicity. The immunotherapy shots can be designed to contain only the essential epitopes necessary for immune modulation, minimizing the risk of triggering severe allergic reactions. Additionally, peptides are less likely to cross-link IgE antibodies, which are responsible for allergic responses. By targeting specific immune pathways, peptides can induce immune tolerance more selectively and with fewer adverse effects than whole proteins. This targeted approach holds potential to enable safer and more effective immunotherapy for allergic conditions, including peanut allergy.

## 2. Results

### 2.1. Logo Model Generation

The logo model consists of two quantitative matrices (QMs)—one for the binders and one for the non-binders. The QMs are focused on the peptide binding core, which is why nonamers were chosen as the training peptides. The QM for the binders was derived from a training set of 105 nonamer peptides binding to HLA-DRB1*03:01 (Table S1). The nonamers were derived from a set of peptide binders to HLA-DRB1*03:01 freely available in NNAlign 2.0 [29]. The peptide data in NNAlign were retrieved from the Immune Epitope Database [30].

A set of 154 non-binders to HLA-DRB1*03:01 was retrieved from the Immune Epitope Database [30] as well. The non-binding peptides were of different lengths, and each peptide was presented as a set of overlapping nonamers, under the presumption that if a peptide is a non-binder, any nonamer derived from it is also a non-binder. Thus, the initial pool of non-binding nonamers consisted of 1187 peptides, a number that decreased to 1123 after eliminating duplicates within the set and those present in the positive set. To mitigate bias in the selection of training and test sets, this final set of non-binders was randomly divided 10 times into a training set of 105 nonamers and a test set of 1018 nonamers. The training sets were used to derive ten QMs for non-binders, and the test sets were used to validate them. The sets of non-binders are given in Table S2.

The amino acid frequencies at each position were mean normalized within the nonamer binding core, according to the following formula:

$$X_{i}\left(norm\right)=\frac{X_{i}-X_{mean}}{X_{max}-X_{min}}$$

where *X_i_* is the frequency of amino acid *i* at a given position, *X_mean_* is the mean frequency for all positions, and *X_max_* and *X_min_* are the maximum and the minimum frequencies, respectively, for all positions. The normalized values are constrained within the range of [–1, 1]. They are arranged into a QM, which measures nine positions of 20 amino acids (Table 1). Amino acids with positive coefficients favor binding to HLA-DRB1*03:01, while those with negative coefficients do not. Amino acids with coefficients around zero are neutral.

**Figure 1:**
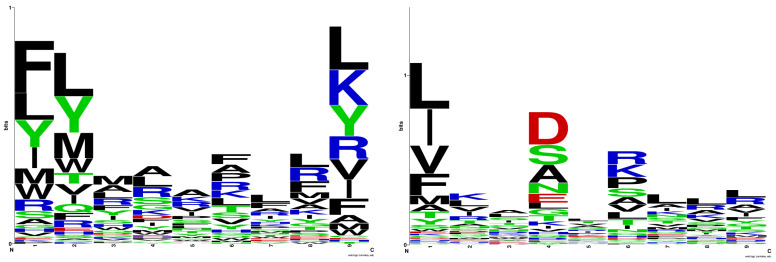
Sequence logo for HLA-DRB1*03:01 derived in the present study (**left**); sequence logo for HLA-DRB1*03:01 according to NNAlign (**right**). The sequence logos were generated by WebLogo (https://weblogo.berkeley.edu, accessed on 23 March 2024).

Figure 1 (left) displays the sequence logo for HLA-DRB1*03:01, generated from the training set of 105 binding nonamers, while Figure 1 (right) illustrates the sequence logo according to NNAlign [29]. There is a strong consensus between the two logos regarding the preferred anchor positions of p1, p6, and p9. The only difference arises in the preferences for the anchor position p4. In the NNAlign logo, the favoured amino acids at p4 are Asp, Ser, Ala, Asn, and Glu. Only Ala and Ser align with the preferences derived from the logo model. This difference could be explained by the composition of the training set used to derive the positive QM of the logo model. Among the 105 binding nonamers, Asp appears in only five peptides, Asn in one, and Glu in two, while Ala, Arg, and Leu each appear in 14 peptides.

Ten QMs were developed based on each training set of 105 non-binding nonamers (Table S2). The QM for the binders was employed to compute the binding score (BS) for a tested peptide by summarizing the values of the corresponding amino acids at each position. Similarly, the QMs for the non-binders were used to calculate the non-binding score (NBS) for a tested peptide. In this case, our interpretation is as follows: amino acids with positive coefficients favor non-binding to HLA-DRB1*03:01, while those with negative coefficients favor binding. Amino acids with coefficients around zero are neutral. If the BS is higher than the NBS, the nonamer is classified as a binder; otherwise, it is identified as a non-binder.

### 2.2. Logo Model Validation

The derived logo model was validated on a test set of binding peptides to HLA-DRB1*03:01 distinct from the peptides included in the training set. The binding peptides were sourced from Immune Epitope Database [30] on 19 February 2024 and contained 10,321 records. After removing internal duplicates and the duplicates present in the training set of the binders, the final test set comprised 7814 binding peptides of different lengths (Table S1). The ten test sets of 1018 non-binders derived as described above were used to validate the corresponding ten QMs for the non-binders derived from the ten training sets (Table S2).

Since the derived QMs cover only the binding core composed of nine residues, binding peptides of varying lengths in the test set were represented as sets of overlapping nonamers. The BS and NBS for each nonamer were predicted using the QMs for the binders and non-binders. If the BS > NBS, the nonamer was classified as a binder; otherwise, it was classified as a non-binder. To classify the parent peptide as a binder, the presence of at least one binding nonamer in the peptide sequence was required.

The validation of the QM for the binders and the 10 QMs for the non-binders is given in Table S2. The sensitivity varied from 0.862 to 0.980, the specificity ranged from 0.628 to 0.707, the accuracy ranged from 0.844 to 0.941, the precision ranged from 0.952 to 0.958, Matthew’s correlation coefficient (MCC) values ranged from 0.451 to 0.689, and F1 scores ranged from 0.907 to 0.967. Among the best-performing QMs for the non-binders, QM1 gave the highest sensitivity, accuracy, MCC, and F1, while QM6 showed the highest specificity and precision but the worst sensitivity and MCC. Based on the highest MCC, we selected QM1 as the best-performing QM for the non-binders. MCC is an efficient predictor estimator given the proportions of positive and negative elements in the datasets [31]. QM1 for the non-binders is given in Table 2. Using all the non-binders as a training set for derivation of a QM for the non-binders did not improve the predictions for the binders or non-binders (Table S2). The results from the validation of the logo model for HLA-DRB1*03:01 are summarized in Table 3.

### 2.3. Prediction of Peptide Binders to HLA-DRB1*03:01 among Peanut Allergens

The information about the peanut allergens was collected from www.allergen.org [4] with the allergen source being *Arachis hypogaea*. Seventeen allergens were found, some with multiple variants. In total, 28 protein sequences were obtained from the UniProt [32] or GenBank [33] databases (Table 4).

Each protein was presented as a set of overlapping nonamers, and the BS and NBS were calculated for each nonamer using the derived QMs for the binders and non-binders to HLA-DRB1*03:01. If the BS > NBS, the nonamer was classified as a binder; otherwise, it was classified as a non-binder. Strong binders were defined as those with a BS greater than two (Table 5).

The predictions of the logo model identified a set of 17 robust binders derived from peanut allergens. A notable relationship exists between strong binders to MHC and T-cell epitopes [34]. The enhanced binding affinity between peptides and MHC augments the probability of efficient T-cell activation, thereby facilitating antigen presentation and subsequent immune responses. With this understanding, it is feasible that these high binders to HLA-DRB1*03:01 could serve as T-cell epitopes and hold promise as potential candidates for allergy immunotherapy.

## 3. Discussion

MHC proteins play a pivotal role in antigen presentation, a fundamental process in adaptive immunity. These intracellular molecules bind peptide fragments derived from pathogens or self-proteins and display them on the surface of antigen-presenting cells for recognition by T-cells. MHC class I molecules present endogenous antigens to CD8+ cytotoxic T-cells, aiding in the detection and elimination of infected or aberrant cells. Conversely, MHC class II molecules present exogenous antigens to CD4+ helper T-cells, orchestrating immune responses by activating various effector cells. MHC-mediated antigen presentation is crucial for immune surveillance and response. Evolutionary pressure shapes the polymorphism of the peptide binding site of MHCs. Diverse MHC alleles increase the range of antigens presented to T-cells, enhancing the immune surveillance of the populations. The highest quantity of the identified MHCs is observed in humans, representing one of the most diverse examples within vertebrates. Presently, the IMGT/HLA database contains structural data on 26,610 HLA class I and 11,398 HLA class II proteins [35], with these figures steadily increasing over time.

HLA polymorphism underlies one’s susceptibility to and protection against infections, autoimmune diseases, and allergenicity. Certain HLA alleles confer a heightened susceptibility to specific infections, while others offer protective effects [36,37]. Moreover, HLA variants are implicated in autoimmune diseases, in which aberrant immune responses target self-tissues [38,39]. Specific HLA alleles are associated with an increased susceptibility or resistance to specific infections. For instance, HLA-B27 is linked to a higher risk of developing severe symptoms from infections like Klebsiella pneumoniae [40]. Conversely, HLA-B57 has been shown to provide some level of protection against HIV progression, as individuals with this allele tend to control the virus better and have a slower disease progression [41]. HLA-DRB1*01:04 is strongly associated with rheumatoid arthritis [42], while HLA-DQB1*06:02 is linked to narcolepsy [43].

The development of allergies is also influenced by HLA polymorphism. Specific HLA alleles can predispose individuals to sensitization to certain allergens. For instance, HLA-DRB1*11 is associated with an increased risk of developing allergies to certain pollens [44], while HLA-DQ2 and HLA-DQ8 are linked to celiac disease [45], a condition triggered by an immune response to gluten. HLA diversity contributes to variations in allergenicity, affecting allergic sensitization and responses [46,47,48,49,50]. The association of peanut allergy to several HLA class II alleles is one of the many proofs supporting the interplay between genetics and immune responses. 

Here, we describe a universal method for the quantitative assessment of peptide–protein interactions based on the amino acid frequencies in the peptide binding cores. The proposed method is not entirely novel. This is why it was named after the sequence logo graphs initially proposed by Schneider and Stephens in 1990 [27]. The novelty of our method lies in the following:

1. Mode of quantification: Schneider and Stephens use Shannon entropy to quantify the nucleic acid/amino acid frequency at a given position. In contrast, we apply mean normalization;
2. Functionality: While the sequence logo method was developed as a graphical tool for visualizing patterns in aligned sequences, our method generates quantitative matrices used to calculate binding and non-binding scores. Based on these scores, peptides are classified as binders or non-binders to a given protein.

We have already applied this method to identify peptides that cause celiac disease [28]. The method is universal and can be applied to quantify various peptide–protein interactions. It holds significant potential and is expected to be utilized by us and other researchers in future studies.

Here, the method was applied to derive a logo model for peptide binding predictions to one specific HLA allele, namely HLA-DRB1*03:01, which is associated with peanut allergy. The QM for the binders to HLA-DRB1*03:01 (Table 1) revealed amino acid preferences at the peptide anchor positions of p1, p4p, p6, and p9. 

**Figure 2:**
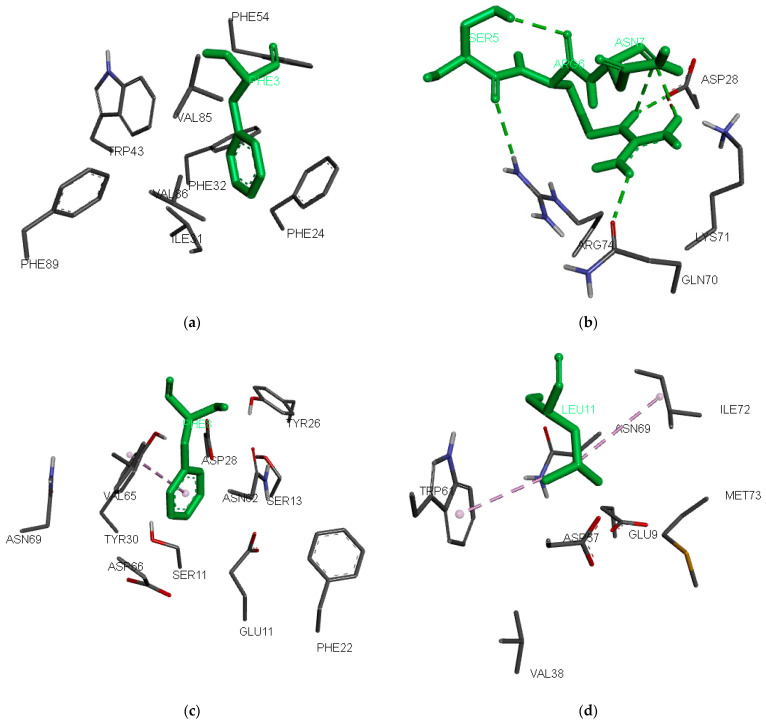
Preferred anchor peptide residues inside the binding pockets: (**a**) 1, (**b**) 4, (**c**) 6, and (**d**) 9. The peptide residues are indicated by green-colored sticks, while the protein residues are indicated by element-colored lines. The docking poses were generated using AutoDock Vina v. 1.2.0 [55]. Hydrogen bonds are indicated with green dashed lines, and hydrophobic interactions are indicated with purple dashed lines.

The most preferred peptide residue in pocket 1 is Phe, followed by Leu, Tyr, and Ile. Pocket 1 stands out as the most crucial pocket in HLA-DR alleles [51,52,53]. It is deep, hydrophobic, and favours residues such as Phe, Trp, Tyr, Leu, Ile, and Met. In HLA-DRB1*03:01, pocket 1 consists of ten hydrophobic residues: Phe24α, Ile31α, Phe32α, Trp43α, Ala52α, Phe54α, Tyr78β, Val85β, Val86β, and Phe89β, and only one polar one: Asn82β (Figure 2a). The composition of the pocket includes the residues within 6 Å around the side chain of p1 in the binding ligand GGI^1^G^2^S^3^D^4^N^5^K^6^V^7^T^8^R^9^RGG from the X-ray structure of HLA-DRA*01:01/HLA-DRB1*03:01 (PDB ID: 7N19; [54]). The Ile at p1 from the original ligand was modified to Phe and flexibly docked to the binding site. Phe (Phe3) comfortably fits into this hydrophobic pocket 1, whereas charged residues like Lys, Glu, and Asp are unfavourable (Table 2).

Binding pocket 4 consists of the residues Thr12β, Ser13β, Glu14β, Tyr26β, Leu27β, Asp28β, Tyr30β, Gln70β, Lys71β, Arg74β, Tyr78β, and Asn82β. It is deep and polar because eleven of the twelve residues are polar or charged. While preferences for negatively charged peptide anchors at p4 are expected, our QM for the binders surprisingly favours Ala, Arg, and Leu. Among the 105 binding nonamers from the training set, Ala, Arg, and Leu each appear in 14 peptides, whereas only five peptides feature Asp and two peptides feature Glu at p4. Notably, the positive electrostatic potential of Arg74β does not deter the binding of Arg in pocket 4. A closer examination reveals that Arg74β forms a hydrogen bond with the backbone oxygen atom of Ser at p3 (Ser5), while Arg at p4 (Arg6) forms three hydrogen bonds: two with the backbone oxygen of Asn at p5 (Asn7) and one with Gln70β (Figure 2b).

At peptide anchor position 6, the most preferred amino acids are Phe, Ala, Arg, and Pro. Pocket 6 is composed of Gln9α, Glu11α, Phe22α, Asn62α, Val65α, Asp66α, Asn69α, Ser11β, Thr12β, Ser13β, Tyr26β, Asp28β, Arg29β, Tyr30β, and Lys71β. Phe (Phe8) fits well here, forming hydrophobic interactions with Val65 (Figure 2c). Arg and Lys also are well accepted in this pocket because of the negatively charged Glu11α and Asp66α.

Pocket 9 is the second most important pocket in HLA-DR alleles. In HLA-DRB1*03:01, it consists of Val65α, Asn69α, Leu70α, Ile72α, Met73α, Arg76α, Glu9β, Tyr10β, Tyr30β, Phe31β, His32β, Glu35β, Asn37β, Val38β, Leu53β, Gly54β, Asp57β, Tyr60β, and Trp61β. The preferences here are for Leu, Lys, Tyr, and Arg. Leu at p9 (Leu11) forms hydrophobic interactions with Trp61 and Ile72 (Figure 2d). Lys and Arg are also considered favourable in this pocket due to the presence of negatively charged Glu9β and Asp57β.

Further, we employed the logo model to analyse the protein sequences of 28 known peanut allergens. This analysis aimed to pinpoint the most probable strong binders to HLA-DRB1*03:01. Our hypothesis posits that these strong binding nonamers may serve as T-cell epitopes. The pathophysiology of peanut allergy involves the participation of peanut-specific CD4+ T-cells. Three T-cell epitopes associated with HLA-DRB1*03:01 originating from Ara h 1, Ara h 2, and Ara h 3 are available in the Immune Epitope Database [30] (Table 6). They contain between four and seven predicted binding nonamers to HLA-DRB1*03:01, with the strongest of them having a BS between 0.363 and 1.437. The measured binding affinities to HLA-DRB1*03:01 are known for only two of them. The peptide ARQQWELQGDRRCQS originating from Ara h 2.0101 is a weak binder (IC_50_ > 500 nM). The best predicted nonamer from this peptide has a very low BS of 0.363. In contrast, the peptide EFLEQAFQVDDRQIV from Ara h 3.0101 is a moderate binder with an IC_50_ of 147 nM, and the nonamer predicted to be the best has a BS of 1.437.

The identification of T-cell epitopes from peanut allergens is crucial for the development of peptide immunotherapeutic agents. There is no definitive evidence indicating whether peptides with strong or moderate HLA binding affinities are more suitable for allergy immunotherapy. It was found that an affinity threshold of approximately 500 nM (preferably 50 nM or less) apparently determines the capacity of a peptide epitope to elicit a CTL response [58]. Strong binders (IC_50_ < 50 nM) are more effective at stimulating T-helper (Th) cells because they bind more tightly to HLA class II molecules and are presented more effectively to these cells. This can potentially lead to a stronger immune response and more effective immunotherapy [59]. However, strong binders can sometimes trigger an overly vigorous immune response, increasing the risk of adverse effects or unintended immune reactions. They might also be less specific, potentially leading to broader or more unpredictable responses [60]. On the other hand, moderate binders (500 nM < IC_50_ < 50 nM) might produce a more controlled and potentially safer immune response. They can still be effective at stimulating Th cells but have a lower risk of overstimulation. This can result in fewer side effects and more manageable therapy. However, they may be less effective at inducing a strong immune response compared to strong binders, which could potentially result in less efficacy in some cases [61].

The choice between strong and moderate binders might depend on how specific the allergen is and how it interacts with the immune system. Strong binders might be favoured for complete desensitization, while moderate binders might be chosen for symptom management. The decision will typically be based on balancing efficacy with safety, as well as considering individual patient factors and therapeutic goals [62].

By administering peptide immunotherapeutic agents in gradually escalating doses, they could potentially desensitize the immune system of patients carrying HLA-DRB1*03:01, thereby increasing their tolerance to peanuts. However, to validate this hypothesis, rigorous clinical trials are imperative. These trials would ascertain whether the identified nonamers indeed induce tolerance and mitigate allergic reactions in patients with peanut allergy. The outcomes of such trials could have profound implications for peanut allergy management, potentially offering a novel immunotherapeutic approach for individuals susceptible to peanut-induced allergic responses. Additionally, understanding the efficacy and safety of this approach would contribute significantly to personalized medicine strategies tailored to specific HLA genotypes. 

## 4. Materials and Methods

### 4.1. Datasets

The positive training set, consisting of binding peptides, was sourced from the freely available peptide dataset in the NNAlign tool [29]. The original dataset comprises 2042 peptides of various lengths binding to HLA-DRB1*03:01. Given the logo model’s focus on the peptide binding core, we specifically chose nonamer binders, resulting in a collection of 105 nonamers.

The test set of binding peptides was collected from Immune Epitope Database [30] with the following settings: Epitope: Linear peptide; Host: Human; Assay: MHC Ligand; Outcome: Positive; and MHC Restriction: HLA-DRA*01:01/HLA-DRB1*03:01. The initial dataset exported on 19 February 2024 contained 10,321 records. Peptide binding affinities were measured either qualitatively or quantitatively by direct or competitive fluorescence assays on cellular or purified MHC, by competitive radioactivity assays on purified MHC, by mass spectrometry on cellular MHC, or by high-throughput multiplexed assays.

The non-binding peptides were collected from Immune Epitope Database [30] with the following settings: Epitope: Linear peptide; Host: Human; Assay: MHC Ligand; Outcome: Negative; and MHC Restriction: HLA-DRA*01:01/HLA-DRB1*03:01. The initial dataset was exported on 1 March 2024, and contained 154 peptides of different lengths. Each peptide was presented as a set of overlapping nonamers, and a pool of 1187 nonamers was generated subsequently and reduced to 1123 after removing the duplicates.

The peptide datasets used in the study are given in Tables S1 and S2.

### 4.2. Statistical Analysis

The performance of the two QMs of the logo model was assessed on the external test sets. True positives (TPs) correspond to peptides correctly identified as binders, while true negatives (TNs) are peptides correctly recognized as non-binders. False positives (FPs) occur when non-binders are incorrectly predicted as binders, and false negatives (FNs) are non-binders incorrectly identified as binders. On this basis, the following measures were calculated:

$$Sensitivity\;(Recall)=\frac{TP}{TP+FN}$$

$$Specificity=\frac{TN}{TN+FP}$$

$$Accuracy=\frac{TP+TN}{TP+TN+FP+FN}$$

$$Precision=\frac{TP}{TP+FP}$$

$$Matthews’s\;correlation\;coefficient\;\left(MCC\right)\;=\frac{TN*TP-FN*FP}{\sqrt{\left(TP+FP)(TP+FN)(TN+FP)(TN+FN\right)}}$$

$$F1\;score=\frac{Precision*Recall}{Precision+Recall}*2$$

### 4.3. Docking Protocol

The X-ray structure of HLA-DRA*01:01/HLA-DRB1*03:01 in complex with the *Aspergillus nidulans* epitope GGI_1_G_2_S_3_D_4_N_5_K_6_V_7_T_8_R_9_RG (PDB ID: 7N19) [48] was utilized in the molecular docking calculations. The original peptide underwent mutation with preferred anchor amino acids, specifically Phe, Arg, Phe, and Leu at positions 1, 4, 6, and 9, respectively, via a single amino acid substitution method. AutoDock Vina v.1.2.0 of AutoDock Suite (Center for Computational Structural Biology, CCSB, La Jolla, CA, USA) [49] was employed for docking, with the following settings: grid box coordinates: x-center: −1.306 Å, y-center: 12.876 Å, and z-center: 15.26 Å; number of points: at x-dimension: 28, y-dimension: 42, and z-dimension 32; standard exhaustiveness (8); and energy range (3). A flexible docking procedure was implemented with fifty-one residues within 6 Å radius from the original peptide in the X-ray structure set as flexible during the calculations. They were Gln9α, Glu11α, Phe22α, Phe24α, Ile31α, Phe32α, Trp43α, Phe51α, Ser53α, Phe54α, Glu55α, Asn62α, Val65α, Asp66α, Asn69α, Leu70α, Ile72α, Met73α, Arg76α, Glu9β, Tyr10β, Ser11β, Thr12β, Ser13β, Glu14β, Tyr26β, Leu27β, Asp28β, Arg29β, Tyr30β, Phe31β, His32β, Glu35β, Asn37β, Val38β, Phe47β, Leu53β, Pro56β, Asp57β, Tyr60β, Trp61β, Leu67β, Gln70β, Lys71β, Arg74β, Tyr78β, His81β, Asn82β, Val85β, Val86β, and Phe89β. During docking calculations, the peptide side chains were flexible while their backbones were kept rigid.

## 5. Conclusions

The peptides identified in the present study offer potential for epitope-based immunotherapy targeting HLA-DRB1*03:01-restricted peanut allergy patients, potentially reducing the risk of irrelevant protein targeting and minimizing undesirable immune responses. Targeted immunotherapy, delivered via subcutaneous injections or orally, allows for gradual immune tolerance induction with personalized dosing. However, clinical validation is essential. Peptide immunotherapy, like any other immunotherapy, has risks including allergic reactions, autoimmune responses, or cytokine release syndrome. Ensuring peptide purity and specificity is crucial to prevent unintended immune activation or off-target effects, while proper handling is essential to maintain therapeutic efficacy and safety. In our opinion, the most suitable peptide candidates for the immunotherapy of peanut allergy have yet to be discovered. Immunoinformatic approaches could serve as valuable tools in this discovery process, helping to identify and predict peptide-HLA binding affinities, T-cell receptor interactions, and potential immunogenicity.

## Figures and Tables

**Table 1:** Quantitative matrix (QM) for binding nonamers to HLA-DRB1*03:01.

aa	p1	p2	p3	p4	p5	p6	p7	p8	p9
Ala	−0.083	−0.157	0.213	0.324	0.324	0.176	0.102	0.028	−0.046
Arg	0.028	−0.046	0.176	0.324	0.176	0.176	0.102	0.398	0.213
Asn	−0.194	−0.194	−0.120	−0.157	−0.009	−0.120	−0.046	−0.194	−0.194
Asp	−0.194	−0.194	−0.083	−0.009	−0.157	−0.194	−0.083	−0.120	−0.194
Cys	−0.157	−0.194	−0.194	−0.157	−0.194	−0.194	−0.194	−0.194	−0.157
Gln	−0.194	−0.009	−0.046	−0.157	−0.120	−0.083	−0.157	−0.120	−0.194
Glu	−0.157	−0.120	−0.194	−0.120	−0.157	−0.194	−0.083	−0.120	−0.157
Gly	−0.120	−0.157	−0.083	0.065	−0.083	−0.083	−0.046	−0.046	−0.194
His	−0.120	−0.120	−0.009	−0.120	−0.157	−0.046	−0.009	−0.120	−0.157
Ile	0.213	−0.009	0.065	−0.009	0.102	−0.194	0.065	−0.009	0.139
Leu	0.324	0.694	0.176	0.324	−0.009	0.139	0.435	0.398	0.583
Lys	−0.157	−0.157	−0.083	0.065	0.176	0.139	−0.009	0.028	0.435
Met	0.102	0.324	0.324	−0.046	−0.046	−0.120	−0.046	0.065	−0.083
Phe	0.806	−0.046	0.102	−0.009	−0.009	0.250	0.250	0.287	0.102
Pro	−0.157	−0.194	−0.120	−0.120	0.065	0.176	−0.009	−0.083	−0.194
Ser	−0.046	−0.120	0.028	0.176	0.065	0.065	−0.083	−0.083	−0.194
Thr	−0.194	0.028	−0.157	−0.157	−0.009	0.102	−0.046	−0.046	−0.157
Trp	0.102	0.102	−0.009	−0.083	−0.083	−0.083	−0.046	−0.120	−0.083
Tyr	0.324	0.546	0.102	−0.009	−0.009	0.028	0.028	−0.009	0.361
Val	−0.120	0.028	−0.083	−0.120	0.139	0.065	−0.120	0.065	0.176

**Table 2:** Quantitative matrix (QM1) for non-binding nonamers to HLA-DRB1*03:01.

aa	p1	p2	p3	p4	p5	p6	p7	p8	p9
Ala	−0.078	0.109	0.234	0.422	0.297	−0.141	0.047	0.359	0.422
Arg	−0.016	−0.078	0.047	−0.141	−0.141	−0.078	−0.016	0.109	−0.203
Asn	−0.078	0.109	0.047	−0.016	−0.078	−0.141	−0.141	−0.203	−0.141
Asp	0.172	−0.016	0.109	−0.078	−0.203	−0.078	−0.203	−0.266	−0.266
Cys	−0.203	0.047	−0.328	−0.016	−0.203	0.047	−0.141	−0.078	−0.016
Gln	−0.141	−0.141	0.109	−0.266	−0.078	−0.203	−0.203	−0.141	−0.141
Glu	0.297	0.047	0.109	−0.078	−0.078	−0.203	0.047	−0.141	−0.016
Gly	0.047	0.359	0.359	0.172	0.109	−0.016	0.297	0.047	0.609
His	−0.141	−0.203	−0.078	−0.141	−0.203	0.109	−0.266	−0.016	−0.141
Ile	0.047	−0.078	−0.016	0.047	0.172	0.047	0.172	0.109	0.047
Leu	−0.078	0.234	−0.016	0.359	−0.016	0.359	0.484	0.422	0.422
Lys	0.297	0.297	−0.078	−0.078	0.109	−0.078	−0.141	0.047	−0.078
Met	−0.328	−0.078	−0.078	−0.016	−0.078	−0.078	−0.203	0.047	−0.141
Phe	−0.078	−0.141	−0.203	−0.016	0.234	−0.141	−0.078	−0.203	0.047
Pro	−0.141	−0.078	−0.203	−0.141	−0.078	−0.266	0.172	−0.016	−0.266
Ser	0.484	0.234	0.234	0.359	0.359	0.672	0.172	0.109	0.234
Thr	−0.141	−0.078	0.234	−0.141	0.047	−0.141	−0.078	0.047	0.172
Trp	−0.328	−0.328	−0.266	−0.266	−0.203	−0.141	−0.266	−0.203	−0.266
Tyr	−0.078	−0.203	−0.328	−0.078	−0.016	0.172	0.047	−0.141	−0.203
Val	0.484	−0.016	0.109	0.109	0.047	0.297	0.297	0.109	−0.078

**Table 3:** Validation of the logo model for prediction of peptide binding to HLA-DRB1*03:01, applying the QM for binders and QM1 for non-binders.

Training set of binders	105 nonamers
10 training sets of non-binders	105 nonamers
Test set of binders	7814 peptides of different length
10 test set of non-binders	1018 nonamers
True positives (TP)	7658
False positives (FP)	365
True negatives (TN)	653
False negatives (FN)	155
*Sensitivity (Recall)*	0.980
*Specificity*	0.641
*Accuracy*	0.941
*Precision*	0.955
*Matthews’s correlation coefficient (MCC)*	0.689
*F1 score*	0.967

**Table 4:** Peanut allergens used in this study [4].

Allergen Name	Allergen and Variants	GenBank Protein	Uniprot
Ara h 1	Ara h 1.0101	AAB00861	P43238
Ara h 2	Ara h 2.0101	AAK96887	-
	Ara h 2.0201	AAN77576	Q6PSU2-1
Ara h 3	Ara h 3.0101	AAC63045	O82580
	Ara h 3.0201	AAD47382	Q9SQH7
Ara h 5	Ara h 5.0101	AAD55587	Q9SQI9
Ara h 6	Ara h 6.0101	AAD56337	Q647G9
Ara h 7	Ara h 7.0101	AAD56719	Q9SQH1
	Ara h 7.0201	ABW17159	B4XID4
	Ara h 7.0301	-	Q647G8
Ara h 8	Ara h 8.0101	AAQ91847	Q6VT83
	Ara h 8.0201	ABP97433	B0YIU5
Ara h 9	Ara h 9.0101	ABX56711	B6CEX8
	Ara h 9.0201	ABX75045	B6CG41
Ara h 10	Ara h 10.0101	AAU21499	Q647G5
	Ara h 10.0102	AAU21500	Q647G4
Ara h 11	Ara h 11.0101	AAZ20276	Q45W87
	Ara h 11.0102	AAZ20277	Q45W86
Ara h 12	Ara h 12.0101	-	B3EWP3
Ara h 13	Ara h 13.0101	-	B3EWP4
	Ara h 13.0102	-	C0HJZ1
Ara h 14	Ara h 14.0101	AAK13449	Q9AXI1
	Ara h 14.0102	AAK13450	Q9AXI0
	Ara h 14.0103	AAT11925	Q6J1J8
Ara h 15	Ara h 15.0101	AAU21501	Q647G3
Ara h 16	Ara h 16.0101	ASU04353	A0A509ZX51
Ara h 17	Ara h 17.0101	ASU04352	A0A510A9S3
Ara h 18	Ara h 18.0101	XP_025675300	A0A444XS96

**Table 5:** Predicted strong binding nonamers to HLA-DRB1*03:01 originating from peanut allergens. Strong binders are defined as those with a BS greater than 2.

Allergen	Predicted Best Binder	BS
Ara h 1.0101	MLLLGILVL	2.102
Ara h 2.0101	LTILVALAL ILVALALFL LVALALFLL FLLAAHASA	2.102 2.620 2.583 2.251
Ara h 3.0101	ALSRLVLRR VLRRNALRR	2.065 2.287
Ara h 3.0201	LLILRWLGL	2.472
Ara h 6.0101	LVALLALVL	2.139
Ara h 10.0101	LLLFAGLAL GLALAGTLL	2.472 2.287
Ara h 11.0101	LLILAGLVL FLASGGFGV	2.731 2.103
Ara h 14.0101	LLLLSGLSL LLLSGLSLL	2.435 2.324
Ara h 15.0101	FLILSGLIL GLIIATPLL	2.880 2.028

**Table 6:** Known T-cell epitopes and predicted strong binding nonamers to HLA-DRB1*03:01 originating from peanut allergens.

Allergen	Known T-Cell Epitope	Binding to HLA-DRB1*03:01 IC_50_ nM	Predicted Best Binder	BS
Ara h 1.0101	NNFG**KLFEVKPDK**KNPQLQD [56]	na	KLFEVKPDK	1.103
Ara h 2.0101	ARQ**QWELQGDRR**CQS [57]	1470	QWELQGDRR	0.363
Ara h 3.0101	E**FLEQAFQVD**DRQIV [57]	147	FLEQAFQVD	1.437

na—not available.

## Data Availability

The original contributions presented in the study are included in the article/Supplementary Materials, further inquiries can be directed to the corresponding author. All datasets used in the study and the derived models are published in this paper.

## Supplementary Materials

The following supporting information can be downloaded at: https://www.mdpi.com/article/10.3390/ph17081097/s1, Table S1: Training and test sets of binding peptides to HLA-DRB1*03:01. Table S2: Training sets, QMs and test sets of non-binding peptides to HLA-DRB1*03:01.

## Abbreviations

BS: binding score; FN: false negative; FP: false positive; HLA: human leukocyte antigen; MCC: Matthew’s correlation coefficient; MHC: major histocompatibility complex; NBS: non-binding score; QM: quantitative matrix; TN: true negative; TP: true positive.
